# An Assessment of Polycyclic Aromatic Hydrocarbons Using Estimation Programs

**DOI:** 10.3390/toxics12080592

**Published:** 2024-08-15

**Authors:** Oluwabunmi P. Femi-Oloye, Ryen T. Sutton, Heidi D. Gordon, Ayush Ain Das, Grace O. Morenikeji, Melissa K. Odorisio, Ovidiu D. Francestscu, Ryan L. Myers, Femi F. Oloye

**Affiliations:** 1 Toxicology Center, University of Saskatchewan, Saskatoon, SK S7N 5B3, Canada; opf5@pitt.edu; 2 Division of Biological and Health Sciences, University of Pittsburgh at Bradford, Bradford, PA 16701, USA; 3 Department of Chemistry, Division of Physical and Computational Sciences, University of Pittsburgh at Bradford, Bradford, PA 16701, USAgordon.heidi24@gmail.com (H.D.G.); rlm166@pitt.edu (R.L.M.); 4 Department of Environmental Science, Division of Physical and Computational Sciences, University of Pittsburgh at Bradford, Bradford, PA 16701, USA

**Keywords:** polycyclic aromatic hydrocarbons, environmental factor, molecular weight, toxicity, biodegradation

## Abstract

In the environment, the class of chemicals known as polycyclic aromatic hydrocarbons (PAHs) behave somewhat differently. This review covers situations where PAHs can be ‘labile’ and where they can be persistent. The in-silico prediction of toxicity and the properties of selected 29 PAHs were estimated using programs developed by the U.S. Environmental Protection Agency (EPA), such as the Estimation Programs Interface (E.P.I.) and the Toxicity Estimation Software Tool (version 5.1.2) (TEST), with online software such as SwissADME and SwissDock. TEST was used to estimate the LC_50_ of the fathead minnow (with a range of 14.53 mg/L for 1-indanone and 2.14 × 10^−2^ mg/L for cyclopenta[c,d]pyrene), the LC_50_ of *Daphnia magna* (with a range of 14.95 mg/L for 1-indanone and 7.53 × 10^−2^ mg/L for coronene), the IGC_50_ of *Tetrahymena pyriformis* (with a range of 66.14 mg/L for 1-indanone and 0.36 mg/L for coronene), the bioconcentration factor (8.36 for 1,2-acenaphthylenedione and 910.1 for coronene), the developmental toxicity (0.30 (−) for 1,2-acenaphthylenedione and 0.82 (+) for 4-hydroxy-9-fluorenone), and the mutagenicity (0.25 (−) for 2-methyl-9-fluorenone and 1.09 (+) for coronene). The carbon chain and molecular weight have a significant effect on the properties of PAHs. Overall, it was found that PAHs with a lower molecular weight (LMW) have a higher water solubility and LC_50_ value and a smaller LogKow value, whereas the opposite is true for heavier PAHs, with TEST predicting that PAHs with an MW of over 168.2 g/mol, with a few exceptions, are mutagenic. Hence, LMW PAHs have a higher potential to be in the environment but are less toxic.

## 1. Introduction

Polycyclic aromatic hydrocarbons (PAHs) are a group of aromatic hydrocarbons having two or more fused benzene rings in various structural configurations [1]. They are hydrophobic and are considered ubiquitous contaminants in the marine environment. These compounds contain only carbon and hydrogen [2]. They are usually found scattered everywhere, including in particles in the air, bodies of water, and sediments through natural and anthropogenic combustion. PAHs having two to four rings are known as having a low molecular weight (LMW) or light PAHs and are more prone to biodegradation as well as to photodegradation, while PAHs with more than four rings are known as having a high molecular weight (HMW) or heavy PAHs and are more persistent. Generally, PAHs have a greater resistance to degradation than many chemicals [3].

LMW PAHs occur in the atmosphere, predominantly in the vapor phase, whereas multiple-ring PAHs (five rings or more) are largely bound to particles. PAHs are harmful organic pollutants, and heavy PAHs, or HMW PAHs, are very hazardous to the environment and human health [1]. They are an abundant group of several hundred chemically related compounds, environmentally persistent with various structures and varied toxicities (Table 1) [4]. Examples of PAHs include naphthalene, acenaphthylene, acenaphthene, fluorine, phenanthrene, anthracene, fluoranthene, pyrene, benz(a)anthracene, chrysene, benzo(b)fluoranthene, benzo(k)fluoranthene, benzo(a) pyrene, benzo(ghi)perylene dibenz(a,h)anthracene, and indeno(1,2,3-cd)pyrene [1].

Atmospheric deposition, oil leakages, and combustion activities are the major sources for the distribution and accumulation of PAHs in water, sediments, and marine organisms. Depending on their source of emission in the environment, PAHs are categorized mainly into two types: petrogenic and pyrogenic PAHs [5]. This classification of PAHs and their sources (natural or anthropogenic) are among the factors determining if they will be labile or persistent in the environment. Petrogenic PAHs are formed through slow, long-term, moderate temperatures, and they arise due to the spillage of crude and refined oil, while pyrogenic PAHs are formed through rapid high-temperature combustion, and they arise due to the combustion of fossil fuels, the incineration of domestic and industrial waste, the burning of biomass, and the production of asphalt.

Previous studies have reported that PAHs of a petrogenic origin are of two or three aromatic rings, while PAHs of a pyrogenic origin are quite often characterized by four–six rings [6]. PAH structures are either linear, as in anthracene; angular, as in dibenz(a,h)anthracene; or clustered, as in pyrene (see Table 1 for PAH structures). The structure of PAHs influences their physicochemical properties [7]. Due to low volatility and long-time persistence, HMW PAHs are reported to have an adverse impact on benthic organisms [8].

Although there are many reviews and reports on PAHs, more knowledge is still needed to help us understand if estimation programs can replace laboratory work or if they are just complementary tools. This review was conducted using the literature and toxicity-assessment software such as TEST, EPI suites, and ADME programs. This review complements existing information that is available and shows how the information derived from predictive software could complement experimental data.A total of 29 selected PAHs and related compounds were used to predict the effects that each chemical can have on various factors relevant to human health and the environment. This paper shows that vital information can be obtained from estimation software; however, caution is needed, as this does not work for all compounds.

## 2. Methods

In this study, TEST (version 5.1.2, https://www.epa.gov/comptox-tools/toxicity-estimation-software-tool-test, accessed on 19 June 2024) was used to predict the LC_50_ of the fathead minnow (96 h), water flea (*D. magna*) (48 h), and rat, orally, the IGC_50_ of the *Tetrahymena pyriformis*, the bioconcentration factor, developmental toxicity, and mutagenicity, following established methods [9,10,11,12].

SMILES (the simplified molecular-input line-entry system) was obtained from the PUBCHEM database and imputed into the estimation software from which results were generated after the software conducted some necessary calculations using the chemical structures. TEST uses many models, but for this research, a consensus model was employed. A consensus model uses the average value predicated from all models within their applicability domain.

EPI Suite™ programs such as KOWWIN™ (estimated log octanol–water partition coefficients), WSKOWWIN™ (estimated solubility), KOAWIN™ (estimated octanol–air partition coefficients), HENRYWIN™ (calculated Henry’s law constants), and KOCWIN™ (estimated organic carbon normalized sorption coefficients for soil and sediment) were used to determine the physicochemical properties of the selected PAHs, according to established methods [12,13]. Structures were drawn using Chemdraw, and a statistical analysis was performed using GraphPad Prism 9 (Boston, MA, USA). The pharmacokinetics and drug-likeness of the selected PAHs were conducted using SwissADME and SwissDock [14,15]. The 4XNN protein receptor (embryo growth hormone receptor in *D. magna*), obtained from the protein data bank, was used for docking.

For the SwissADME (http://www.swissadme.ch/, accessed on 19 June 2024) and SwissDock (SwissDock 2) programs (https://www.swissdock.ch/results.php?job=90443651, accessed on 19 June 2024), the SMILES for each of the chemicals was entered individually into the search bar under submit a ligand, and then the ligands were prepared by clicking on the necessary button, after which the protein (4XNN) was prepared. “Select all” was selected to keep chains: A-cellulose 1,4-beta-cellobiosidase (the non-reducing end) and B-cellulose 1,4-beta-cellobiosidase (the non-reducing end). No heteroatoms were kept. Once the target was prepared, the search space was not modified, and the suggested search space was used. Under “Select parameters” the number of Random Initial Conditions (RICs) was set to one. After the docking was complete, under the “Best members” tab, the cluster number that was selected was zero, and the cluster member selected was one.

## 3. Results and Discussion

All the prediction software used in this study could generate the chemical formula, MW, and structural formula. Nevertheless, the structures presented in Table 1 were generated using CHEMDraw. All structures have at least two aromatic rings, except for indanone. The lightest PAH is naphthalene with just two aromatic rings, and as the MW increases, the number of attachments in the structure increases, which is expected to affect their activities. Compounds similar to PAHs in the structure but with heteroatoms were referred to as PAH-related compounds, and some examples of these are 1-Indanone, 1-Acenaphthenone, 1,2-Acenaphthylenedione, 9-Fluorenone, 2-Methyl-9-Fluorenone, 1,8-Naphthalic anhydride, and 4-Hydroxy-9-Fluorenone (Table 1). The presence of heteroatoms, such as oxygen, make the pattern between the MW and structural formula observed in PAHs different from that observed in PAH-related compounds. Knowing the structure and molecular formula of compounds is very important in understanding the chemical properties of compounds. The structural formula reveals all the elements present in compounds as well as how they are connected.

## 4. PAHs, Their Physicochemical Properties, and Implications

PAHs have natural as well as anthropogenic sources. They are widespread environmental pollutants that are formed in the combustion process of carbonaceous materials at high temperatures. The lighter the PAH, the greater the possibility that the PAH will be found in the environment. Thus, since Naphthalene, with a MW of 128.17 g/mol, is lighter than high-MW PAHs, such as coronene (with a MW of 300.4 g/mol), the former has a higher potential for being found in the environment than the latter. It has been reported that Naphthalene is the most abundant PAH in the Amazonian rainforest in Brazil, with over 85% of it being present in the air, plants, and litter and greater than 55% in termite nests [16]. Another report concluded that the mobility of PAHs is the major reason for this, while the PAH concentration in the atmosphere varies [17]. The major source of exposure to PAHs in the general population is from breathing contaminated ambient indoor air. This contamination does not only come from the infiltration of outdoor air but also from indoor emission sources such as cooking, smoking, domestic heating with fuel stoves, the consumption of barbecued food, open fireplaces, and from incense and candle emissions. Exposure to PAHs can also be occupational, occurring from workers breathing exhaust fumes such as those emitted from street vendors, motor vehicle mechanics, and those involved in mining and metal working. Thus, in a PAH-polluted environment, everything precipitating from the atmosphere will be contaminated. Krauss et al. [16] concluded that the atmosphere is the major source of all PAHs in plants. A major route of exposure is the consumption of food, and, for smokers, the contribution from smoking can be an additional component [18]. Food can be contaminated from environmental sources (natural and mostly anthropogenic), from industrial food processing, and from domestic cooking practices [19]. PAHs can enter the food chain by deposition from the air or by disposition and transfer from soil and water [1,18]. High amounts of LMW PAHs, such as naphthalene, acenaphthylene, and acenaphthene, have been found in fruits [18]. PAHs are widespread environmental pollutants, so widespread that it is impossible for anyone to avoid exposure to them [1]. Exposures to PAHs may involve more than one route simultaneously, affecting the total absorbed dose.

The fate of PAHs, their chemical properties, and the environmental fate of PAH molecules are dependent, in part, upon both molecular sizes, i.e., the number of aromatic rings, and the pattern of ring linkage [20]. Most PAHs occur as hybrids encompassing various structural components, such as in the PAH benzo[*a*]pyrene (B*a*P) (Table 1). Generally, an increase in the number of rings and angularity of a PAH molecule results in a related increase in their water-resistant capacity and electrochemical stability [21]. The two primary factors which contribute to the persistence of HMW PAHs in the environment are molecule stability and hydrophobicity [18]. The MW and structure can help to understand if a chemical will be stable in a particular matrix or not. As the MW of the PAH increases (Table 1), the LogKow (octanol–water partition coefficient) increases and the solubility decreases (Table 2), which indicates that these properties are very important in understanding the transportation of PAHs. PAH concentrations in the environment vary widely, depending on the proximity of the contaminated sites to the production source, the level of industrial development, and the mode(s) of PAH transport [18]. The modes of transport of PAHs include stormwater runoffs, sediment-facilitated transport, air transport, and so on. The common characteristics of PAHs are high boiling and melting points (therefore, they are solid); low vapor pressures; and very low aqueous solubilities [22]. The last two characteristics tend to decrease with an increasing MW, while the resistance to oxidation and reduction increases [22].

Kow is an n-octanol/water partition coefficient, and LogKow is used to predict the potential of a compound to be in the organic phase or aqueous phase. There is a strong correlation (r = 0.98, *p* < 0.0001) between the predicted LogKow and the experimental value obtained from the EPI Suites™ library and the literature [23], confirming that KOWWIN™ can be used to estimate the LogKow of PAHs when experimental data are not available. Nevertheless, our earlier study with an herbicide (furilazole) and a safener (benaxacor) showed that KOWWIN™ overestimates the LogKow by 19% and 24%, respectively [13]. It is important to note that the trend of PAH LogKows was different from that of PAH-related compounds due to the presence of an oxygen atom within their molecules. The presence of oxygen might affect the calculation performed by the programs.

While the prediction was nearly similar to experimental data for density, there was huge difference between the experimental and predicted values for solubility. As an example, the density of naphthalene was 1.03 g/mL and 1.04 g/mL for the predicted and experimental, respectively, and its solubility was 142.1 and 31 mg/L for the predicted and experimental, respectively, indicating that the program was more accurate for density calculation than solubility.

LogKoa (octanol–air partition coefficient) followed the same pattern as LogKow, which means that LMW PAHs are prone to be more available in the air of contaminated sites. PAHs also demonstrate a variety of properties such as light sensitivity, heat resistance, conductivity, the ability to emit, corrosion resistance, and physiological actions [4]. Interestingly, LogKoa had a strong correlation of 1, *p* < 0.0001, when the available experimental data were compared with the predicted LogKoa. Thus, predicted data can be used when experimental data are not available.

Another useful parameter to determine the abundance of a volatile solute in a liquid proportional to its abundance in the gas phase is Henry’s law constants. These are not ideal for comparing the distribution of a chemical between a solid (soil or sediment) and water [24,25]. Hence, it is better to use octanol–gas partition coefficients. LogKoa and Henry’s law constants are inversely proportional. Similarly, as the MW increases, the Henry’s law constants decrease.

The Henry’s law constants were obtained from Henry’s law experimental databases or were generated using the SAR Group method* and the Bond SAR method+ when no experimental data were present. PAHs can be introduced into aquatic ecosystems through atmospheric deposition, wastewater discharges, navigation activities, and oil spills. In consideration of their hydrophobic properties, PAHs tend to adsorb to other particles and deposit around contaminated sites. Therefore, the compound of a PAH that is more hydrophilic will be water-loving and mobile, compared to a hydrophobic one, which will not be mobile and thereby will attach to soil or sediments. In some favorable conditions, adsorbed PAHs can be released into water and become bioavailable in an aquatic ecosystem [26]. Bioavailability is a major source of variability in persistence data.

PAHs have been reported in many places because of their widespread presence in the environment. The overall mean of PAH concentrations reported in a Northwestern coastal marine environment of India was reported to be significantly higher than the mean PAHs in Colombia, South Africa, and Egypt [6]. In one study, HMW PAHs accounted for 80.50% of the PAHs found in surface water samples. Another study confirmed that HMW PAHs were dominant, with 42% being present in water and other in sediments [27]. In another study, the calculated ratio values of LMW/HMW PAHs in water samples from five points were less than one [28]. This may be due to the low solubility of HMW PAHs in water, the less volatility being due to their molecular size and higher persistence in an aqueous environment when compared to LMW ones. A major source could be linked to anthropogenic activities rather than natural sources. Interestingly, Grmasha et al. [27] observed that PAH contamination increases along the flow direction, which is due to non-point source pollution and agriculture. Nevertheless, the diagnostic ratio of PAHs revealed that the measured PAHs originate from the combustion of petroleum products [27]. The PAH concentration was reported to be relatively high in the river region in the river mouth of Southern Kaohsiung in Taiwan, then gradually diminishes towards the harbor region [29]. This indicates that PAHs are predominantly from pyrogenic sources as a result of an increase in the concentration of HMW PAHs over LMW PAHs, thereby reducing the LMW/HMW ratios to below 1. The reason for the dominance of HMW PAHs in the surface water in this study could be attributed to the persistence of HMW PAHs in the sediments, which get released back into the aquatic environment, or to the closeness of the sampling points to the source of the PAHs. The overall result suggests that pyrogenic sources including the combustion of fossil fuels and vehicle emissions are responsible for the permission of PAHs in the study area [6]. Since the aqueous solubility of PAHs decreases for each additional ring, coupled with other characteristics including a low vapor pressure, the HMW PAHs observed in this study are, therefore, found in the surface water and are not quite available for subsequent degradation. This observation agrees with the information in Table 2, which shows that LMW PAHs have lower LogKow values and are, therefore, mobile, whereas HMW PAHs have higher LogKow values and thereby are more hydrophobic, hence the possibility of being detected near the source of pollutants.

Edokpayi et al. [28] also showed that the total PAHs in the Mvudi river was higher than those in the Nzhelele rivers, and the concentration of PAHs were higher in the sediments of both rivers than their water samples. This variation can be attributed to the hydrophobic nature of PAHs, as they tend to adsorb on the surface of sediments, because they are not soluble in water. Generally, HMW PAHs with ≥four rings were predominant in the river, sediment, and WWTF samples. Using diagnostic ratios, coupled with LMW/HMW ratios with values of <1, the source of PAHs from these rivers were pyrogenic sources due to the combustion of bushes and other biomasses. Hence, a careful analysis of the levels of LMW PAHs and HMW PAHs, coupled with an understating of their octanol–water partition coefficient, is essential to understand their sources.

As stated earlier, the atmosphere is the most important means of PAH dispersal, as it receives the bulk of the environmental load of PAHs, resulting in PAHs being universally present in the environment. The knowledge of their partition in octanols and the air will help in knowing their abundance in the air and their probability of precipitating to soil, water, and plants. The LogKoa increases as the MW increases; thus, HMW PAHs have a higher probability of precipitating, while LMW PAHs have a higher probability of staying longer in the air and, therefore, transport to a more disperse environment, far away from the source of PAHs. Hence, LogKoa can help explain the abundance of LMW PAHs reported by Krauss et al. [16]. PAHs released into the atmosphere are found in two separate phases, a vapor phase and a solid phase, in which the PAHs are adsorbed onto particulate matter (aerosols).

Furthermore, the combination of LogKoa and LogKow can help improve our understanding of the sources of PAHs. Environmental distributions of PAH constituents in the air are based on their physical and chemical characteristics. For example, vapor- and dissolved-phase PAH distributions in the air, precipitates, and water are dominated by two- and three-ringed species (LMW PAHs), whereas aerosols and particulate phases and sediments are generally dominated by four-, five-, and six-ringed species (HMW PAHs), which are typical of pyrogenic sources [30], making HMW PAHs more persistent and less bioavailable. The removal of PAHs from the atmosphere involves dry and wet deposition processes and is strongly influenced by their gas/particle partitioning. Atmospheric deposition is a major source for PAHs in soil [4].

Hydrophobic organic chemicals with low vapor pressures, such as PAHs, are adsorbed by atmospheric particulates more readily than chemicals with high vapor pressures, such as benzene. The inconsistency in vapor pressures of different PAH compounds cause each PAH to distribute, in different concentrations, in the vapor and other adsorbed phases [4]. A positive correlation was established between the vapor pressure and MW of PAHs [4]. Therefore, the relationship between the MW and vapor pressure of PAHs is a factor that could point towards the distribution and fate of PAHs. PAHs with lower vapor pressures (e.g., benzo[a]pyrene) tend to attach to particles, while PAHs with higher vapor pressure (e.g., naphthalene) tend to associate with the vapor phase [4]. As a result, the relative distribution of PAHs and the two phases would be different for an air sample. PAHs attached to the vapor phase can be easily broken down or changed by several natural factors, while those attached to particles tend to persist longer. For example, air samples collected from Portland, Oregon by the Electric Power Research Institute showed that there are two differences between the PAH concentrations in the particulate and vapor phases [31]. Firstly, the total PAH concentration for the vapor phase was much higher than that of the particulate phase. Secondly, LMW PAHs and PAHs with a higher vapor pressure were detected in the vapor phase, whereas PAHs with a HMW and lower vapor pressure were not. In contrast, the vapor phase has a much lower concentration of HMW PAHs than that of the particulate phase [31].

Humidity also influences the adsorption of PAHs onto particulate phases, because an increase in the relative humidity leads to an increased polarity of PAHs, which could enhance the bioavailability of such substances. Moreover, the adsorption of PAHs also depends on the types of suspended particulates (e.g., soot, dust, pyrogenic metal oxide fly ash, pollen, and so on) [32].

On the other hand, temperature is a factor that will also affect the rate at which PAHs are deposited from the atmosphere [4]. Higher temperatures will cause a greater portion of the total PAHs to be in the vapor phase, and lower temperatures will increase the sorption of PAHs [33]. For example, one study found that about 93% of naphthalene was found in the vapor phase in the air sampled in the summer, while only about 53% of naphthalene was in the vapor phase in the air sampled during the winter [34]. This shows the effect of a high temperature on naphthalene during the summer as compared to the low temperatures during the winter. The higher PAH level in the winter can be ascribed to the high amount of fossil fuels that have undergone incomplete combustion, elevated residential heating, lower photodegradation, and poor diffusion due to atmospheric conditions like calm winds and low temperatures [35]. Thus, knowing the LogKoa value for each PAH, in combination with environmental factors, is essential to knowing the fate of PAHs in each environment.

### PAHs and Their Mobility in Soil [4,6,27,28]

When PAHs precipitate from the air, they either reside in water, sediments, or soils. Hence, it is important to understand their mobility in soil. Koc is a soil adsorption coefficient, and LogKoc is useful in predicting the mobility of compounds in soil. The organic carbon sorption/partition coefficient is directly proportional to Kow (Table 2), since the Pearson correlation coefficient between LogKoc and LogKow is greater than 0.96, regardless of the method for calculating Koc. The soil adsorption coefficient increases with the MW; therefore, it will have an effect similar to LogKow. Nevertheless, the predicted values with the MCI method are higher than the experimental values (by about twice the amount with the Kow method) (Table 2). These observed differences could be related to the method of calculation, since the MCI method utilizes molecular connectivity as fragments; contiguous bonds, whether they are pi, sigma, or lone-pair electrons; and the recombination of such fragments to craft an algorithm, while the Kow method relies on calculated octanol–water partition coefficients. A high LogKoc indicates strong adsorption onto soil and organic-matter compartments.

Since a majority of PAHs are attached to soil particles, important factors influencing the mobility of PAH particulates in the subsurface are the particle size, pore size, and pore volume of the soil [4]. If the particulate matter to which PAHs are bound cannot move through the soil, then there will be limitations in the movement of PAHs, because they partially remain attached to particles. Thus, the fate of PAHs in soil or sediments and the tendency to be bound to soil depends on the properties of the soil and that of the PAHs. Therefore, sorption of PAHs is one of the many processes that control the soil mobility of individual PAHs. Several studies have correlated the partition coefficient with soil properties and have discovered that the organic carbon content usually yields the most significant correlation [31]. The octanol–water partitioning coefficient is related to the solubility of an organic compound in water. An increase in the Kow value results in a decrease in the aqueous solubility and an increase in the tendency for sorption to a particular soil [4]. Nevertheless, solubility and Kow can affect the mobility of PAHs in soil and in turn affect their persistence. Soluble/highly vaporised PAHs can be labile and less persistent compared to less soluble ones—usually, HMW PAHs.

The age of PAHs in the soil and differences in the desorption rate over time also affect how rapid desorption from soil will be in order to be less persistent [36]. When the resident time of PAHs in the soil increases, the desorption rate of the pollutants decreases [30]. Movements of PAHs through the soil/sediment affects their degradation [37]. Thus, understanding LogKoc is important to understanding the fate and bioavailability of PAHs. Other factors, such as soil conductivity, also play a significant role in PAH movements.

Soil has an abundance of microorganisms, which can affect the degradation of PAHs in soil [20,38]. PAHs can become less persistent when they undergo biodegradation/ bacteria bioremediation. For bacteria to degrade in any given PAH, such a PAH must be made available for uptake by the bacteria. These compounds become bioavailable when they are in the vapor or dissolved phase. Therefore, sorption of PAHs to soil is an important factor when considering biodegradation, because PAHs that adsorb onto soil particles cannot be readily degraded by bacteria, because the PAHs are separated from the enzymes that are used by the bacteria to break them down [39]. A study was conducted in the Fujian province of China, where sediment samples were collected from a mangrove area in Fugong. A total of 53 strains of PAH-degrading bacteria were isolated from the mangrove sediments, consisting of strains of phenanthrene, pyrene, and benzopyrene and mixed strains of PAH-degrading bacteria. The results show that both the phenanthrene and mixed-strain PAH-degrading consortia had the highest ability to degrade the phenanthrene in a liquid medium, with more than 91% being degraded in three days [8]. These results suggest that a higher degradation of PAHs is dependent on both the bacteria consortium present and the type of PAH compound.

PAHs have a high potential for biomagnification through trophic transfers due to their lipophilic nature [40]. The relationship between the environmental persistence of PAHs and the increasing number of benzene rings has been reported to have an association with correlating rates of environmental biodegradation and the molecule size of PAHs [20]. For example, as reported in a study, half-lives in the soil and sediment of the three-ringed phenanthrene molecule may range from 16 to 126 days, while for the five-ringed molecule B*a*P, they may range from 229 to >1400 days [41]. Thus, more rings lead to a higher persistence within the environment.

Other forms of degradation methods that make PAHs less persistent and bioavailable include chemical methods like soil washing with solvents, a suitable approach to removing HMW PAHs, which are difficult to remove from soil due to their strong affinity to soil and low bioavailability [42]. Soil washing can be used alongside other methods for maximum degradation. Surfactant use may enhance the efficiency of soil washing by changing the solubility of PAHs. This process is strongly dependent on the properties of PAHs, soil compositions, and surfactant structures. Suitable solvents or a mixture of solvents can also be used to remove PAHs from sludge, water, and soil [43]. In summary the mobility of PAHs in soil, which is governed by LogKoc, plays a significant role in the fate and bioavailability of PAHs with respect to soil microorganisms [4,20,29,41].

PAHs can also become labile when photodegraded. This is a process of destroying a compound from reactions initiated by the absorption of light. This reaction occurs when light absorbed by PAHs excite an electron within the molecule. This excitation creates an unstable structural arrangement. As a result of this, such an unstable structural arrangement allows for several natural processes to act on the excited PAHs [4]. Photodegradation reactions depend strongly on the structure of PAHs, which affect their ability to be degraded. For example, some linear two rings and some clustered PAHs degrade rapidly under direct sunlight while angular PAHs (e.g., phenanthrene and dibenze[a,h] anthracene) take a long time to degrade, because they are the most structurally stable molecules [44]. LMW PAHs are more prone to photodegradation, because these compounds are more bioavailable and have longer exposure times to sunlight [4].

A study was carried out to measure the photolysis kinetics of the PAHs anthracene and pyrene in several organic solvents and in water, as well as in miscible and phase-separated aqueous organic mixtures at atmospherically relevant wavelengths [45]. The results show that constants of the photolysis rate generally increased with an increasing solvent polarity; photolysis was more than 10 times faster in the water than in octanols. The quick degradation of the three- and four-ringed compounds in the water could be linked to their LMW, which makes them available for degradation, unlike nonpolar octanols [45].

## 5. Toxicity [40]

**Figure 1:**
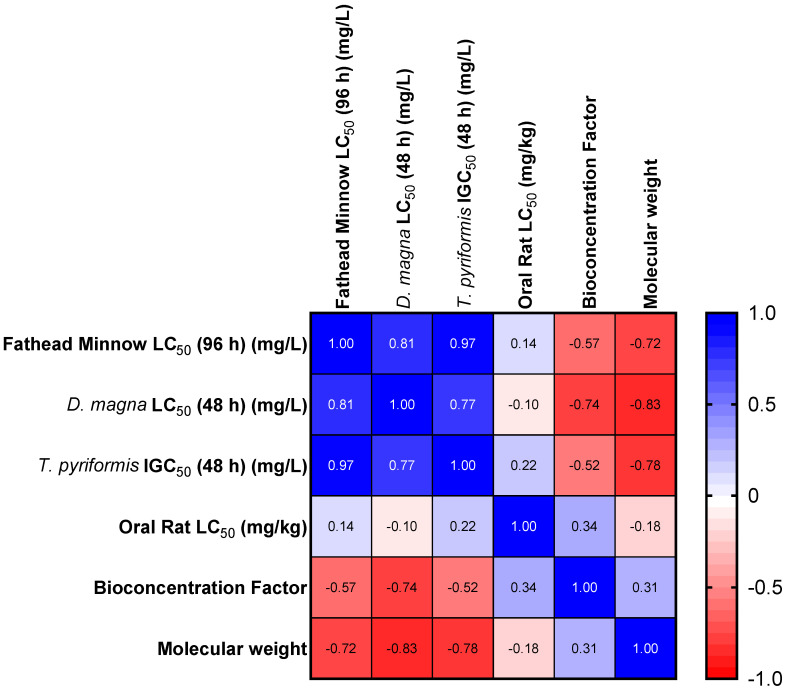
Heat map for the Spearman correlation of selected endpoints and MWs.

PAHs are generally harmful pollutants, but recently, some of these pollutants were found to be biodegraded, thereby making them less persistent and bioavailable in the environment [38,46]. A majority of these less-persistent ones are those with LMWs. They are usually generated in a broader temperature range (petrogenic) than HMW PAHs, which are formed from rapid high-temperature combustion and are, therefore, referred to as pyrogenic. The toxicity of PAHs increases as the MW increases for the Fathead minnow, *D. magna*, and *T. pyriformis*. However, there is no direct relationship between the toxicity and MW for rats in predicted oral exposures (Table 3). The correlation between the LC_50_ (or IGC_50_) and MW is less than one (Figure 1), suggesting that the higher the LC_50_, the lower the MW and the lower the toxicity. The number of rings in PAHs also determines their toxicity [28]. Similarly, the orientation of the aromatic ring (or their arrangement in space) affects the toxicity of compounds. For instance, benzo[b]fluoranthene, benzo[k]fluoranthene, benzo[j]fluoranthene, benzo[a]pyrene, benzo[e]pyrene, and perylene all have the same MW but different LC_50_ values (for the Fathead minnow and *D. magna*), bioconcentration factors, development toxicities, and mutagenicity factors (Table 3).

Differences between the experimental and predicted data could be a result of errors during the experiments or because of over/under estimations by predictive programs. Using naphthalene as an example, the predicted LC_50_ value of the Fathead minnow was 7.14 mg/L, while the experimental value was 6.14 mg/L. The D. magna LC_50_ value was predicted to be 7.42 mg/L, while the experimental value was 9.14 mg/L. This indicates discrepancies between the predicted and experimental values, but both datasets exhibit the same overall trend. Therefore, it is crucial to consider the trends of chemicals when using predictive and experimental data.

Generally, the chain length and MW is expected to play a key role in the bioconcentration factor, owing to steric hindrances, and this is somehow true, because naphthalene has a lower bioconcentration factor compared to other compounds; however, the correlation between the MW and bioconcentration factor is less than 0.4. Other endpoints, such as developmental toxicity and mutagenicity, do not have a direct relationship with the chain length and MW. Hence, in addition to the chain length and MW, the number of aromatic rings, their orientation, and the presence of heteroatoms affect the toxicity of compounds.

As an example of oxygenated PAHs, 9-fluorenone was less toxic compared to fluorene in a simulated acute toxicity study, even when both had the same carbon length (C_13_) (Table 3). This difference in toxicity could be attributed to the additional functional group in 9-fluorenone. Nevertheless, in a chronic exposure, the oxygenated compound has a higher bioaccumulation factor. These observations suggest that the presence of heteroatoms in compounds can completely change the toxicity of PAHs. The mutagenicity positivity and developmental toxicity did not follow a particular trend; thus, the number of rings or heteroatoms affect these properties more.

It is important to note that the toxicity of PAHs correlated well in the three model organisms (*T. pyriformis*, *D. magna*, and Fathead minnow). This correlation might be related to the position of each of the model organisms in the food chain. The correlation between oral rat LC_50_ values was weak compared to any of the acute endpoints in any of the organisms (*T. pyriformis*, *D. magna*, and Fathead minnow). 

SwissDock was used to understand the interaction of selected PAHs and the 4XNN protein (Table 4). The SwissParam score provides an estimate of the binding free energy as a weighted sum of the polar and nonpolar terms [45,46,47,48]. The SwissParam score is provided to compare multiple ligands for the same target. Dibenzo[a,h]anthracene had the lowest SwissParam score of −6.9921. Naphthalene had the highest SwissParam score of −5.8241. 1-Indanone had the lowest AC score of 4.5814, and Indeno[1,2,3-cd]pyrene had the highest AC score of 60.0970. These PAHs and related compounds seem to sufficiently interact with the 4XNN protein via hydrophobic contacts.

SwissADME was used to understand the interaction of PAHs within living cells and their drug-likeness (Table 5). Lipinski value was “Yes”, and the bioavailability score was 0.55 (55%) for each PAH. This indicates that all the PAHs and the related compounds met the drug-likeness criteria and interacted with host cells. Since all PAHs follow Lipinski’s rule of 5 and have a bioavailability score of 0.55, these PAHs have ideal absorption in the body. Nevertheless, gastrointestinal absorption was low for all PAHs, except Anthracene, and high for all the related compounds, which is due to the presence of heteroatoms in the related compounds. An earlier report showed that many PAHs are readily absorbed from the gastrointestinal tract of mammals, because they are highly lipid soluble [4]. While none of the PAHs or related compounds inhibit CYP2C9, CYP2D6, and CYP3A4, all of them are CYP1A2 inhibitors, suggesting that these chemicals might be organ specific. Generally, the PAH metabolism was believed to occur through a cytochrome P450-mediated mixed-function oxidase system with oxidation or hydroxylation [4].

Since PAHs and related compounds have drug-likeness properties, they could potentially reduce the effectiveness of medications when present in the body; thus, no amount of PAHs should be present in drinking and domestic water or in water of economic importance.

## 6. Conclusions

Programs within EPI Suites, TEST, and SwissADME were used to predict the physicochemicals and toxicity of PAHs and related compounds. The results show that the LogKow, LogKoa, and LogKoc correlated well with trends reported from experimental data. The MW, structure, and nature of the elements in the compounds are a key factor that affects their properties. As the MW increases, the LogKow increases and the mobility of PAHs in an aqueous environment decrease. LMW PAHs have lower LogKow values and are, thereby, more hydrophilic than HMW PAHs; hence, they are more abundant in areas far away from their source of pollution. Also, the MW of PAHs can help identify the source of the PAHs. HMW PAHs are more persistent than LMW PAHs in the environment due to their increased resistance to oxidation, reduction, and vaporization compared to LMW PAHs. LogKoc and LogKoa helped to understand the abundance of PAHs in the air and in soil. LMW PAHs have a higher tendency to be abundant in the air, while HMW PAHs have a higher tendency to precipitate. The number of their rings and their arrangements were found to be another significant factor that affects physical and chemical properties. As the MW increases, the solubility of PAHs decreases and a slight increase in the density becomes apparent. Solubility, LogKow, LogKoa, and LogKoc all play key roles in the mobility and bioavailability of PAHs. Except for solubility, the EPI Suite programs have results which correlate well with experimental values.

The TEST results show that the higher the MW, the higher the toxicity. The toxicity results for *T. pyriformis*, *D. magna*, and Fathead minnow might be related to their levels in the food chain. The presence of oxygen in PAH-related compounds greatly affects their toxicity. The orientation of aromatic rings also affects their toxicity; thus, in addition to their MW, the structure of PAHs affects their toxicity. ADME predictions showed that all PAHs were drug-like and could potentially alter the host cells. Nevertheless, none of them inhibit CYP2C9, CYP2D6, and CYP3A4. Gastrointestinal absorption was low for all PAHs, except Anthracene, and high for all the related compounds, confirming that PAHs and PAH-related compounds behave differently.

The SwissParam scores showed that all PAHs and related compounds interact with 4XNN through hydrophobic contacts. This confirms that both PAHs and related compounds can interact with host cells.

## Figures and Tables

**Table 1:** The MW and structural formula of PAHs and related compounds.

PAH	Formula	MW (g/mol)	Structural Formula
Naphthalene	C_10_H_8_	128.2	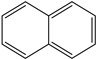
Acenaphthylene	C_12_H_8_	152.2	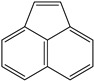
Acenaphthene	C_12_H_10_	154.2	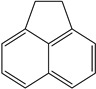
Fluorene	C_13_H_10_	166.2	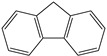
Phenanthrene	C_14_H_10_	178.2	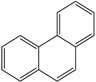
Anthracene	C_14_H_10_	178.2	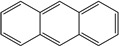
Fluoranthene	C_16_H_10_	202.3	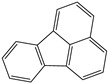
Pyrene	C_16_H_10_	202.3	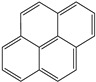
Cyclopenta[c,d]pyrene	C_18_H_10_	226.3	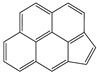
Benzo[a]anthracene	C_18_H_12_	228.3	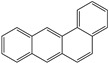
Chrysene	C_18_H_12_	228.3	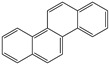
Benzo[b]fluoranthene	C_20_H_12_	252.3	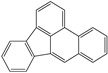
Benzo[k]fluoranthene	C_20_H_12_	252.3	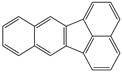
Benzo[j]fluoranthene	C_20_H_12_	252.3	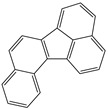
Benzo[a]pyrene	C_20_H_12_	252.3	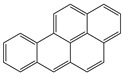
Benzo[e]pyrene	C_20_H_12_	252.3	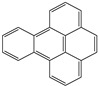
Perylene	C_20_H_12_	252.3	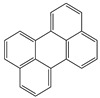
Indeno[1,2,3-cd]pyrene	C_22_H_12_	276.3	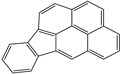
Dibenzo[a,h]anthracene	C_22_H_12_	276.3	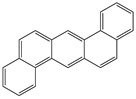
Benzo[b]chrysene	C_22_H_14_	278.3	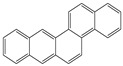
Benzo[g,h,i]perylene	C_22_H_12_	276.3	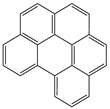
Coronene	C_24_H_12_	300.4	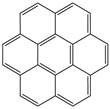
1-Indanone	C_9_H_8_O	132.2	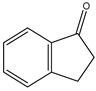
1-Acenaphthenone	C_12_H_8_O	168.2	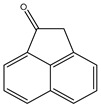
1,2-Acenaphthylenedione	C_12_H_6_O_2_	182.2	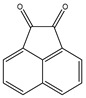
9-Fluorenone	C_13_H_8_O	180.2	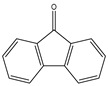
2-Methyl-9-Fluorenone	C_14_H_10_O	194.2	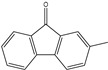
1,8-Naphthalic anhydride	C_12_H_6_O_3_	198.2	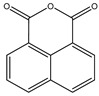
4-Hydroxy-9-Fluorenone	C_13_H_8_O_2_	196.2	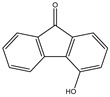

**Table 2:** Physicochemical properties of PAHs and related compounds as determined from TEST and EPI Suites.

PAH	LogKow (Pred.)	LogKow (Exp.)	Solubility (Pred.) (At 25 °C (mg/L))	Solubility (Exp.) (At 25 °C (mg/L))	Density (g/cm^3^) (Pred.)	Density (g/cm^3^) (Exp.)	LogKoa (25 °C) (Pred.)	LogKoa (25 °C) (Exp.)	LogKoc (MCI)	Koc (Kow)	Henry’s Law Constant (atm-cm^3^/mol)
Naphthalene	3.17	3.30	142.10	31.00	1.03	1.04	5.05	5.19	1544.00	730.60	4.40 × 10^−4^
Acenaphthylene	3.94	3.94	2.49	16.10	1.16	1.19	6.27	N/A	5027.00	2625.00	1.14 × 10^−4^
Acenaphthene	4.15	3.92	2.53	3.90	1.11	1.07	6.04	6.31	5027.00	2522.00	1.84 × 10^−4^
Fluorene	4.02	4.18	1.34	1.69	1.08	1.12	6.59	6.79	9160.00	4241.00	9.62 × 10^−5^
Phenanthrene	4.35	4.46	0.68	1.15	1.12	1.13	7.22	7.57	1.67 × 10^4^	7421.00	4.23 × 10^−5^
Anthracene	4.35	4.45	0.69	0.04	1.13	1.13	7.09	7.55	1.64 × 10^4^	7274.00	5.56 × 10^−5^
Fluoranthene	4.93	5.16	0.13	0.26	1.24	1.25	8.60	8.88	5.55 × 10^4^	3.01 × 10^4^	8.86 × 10^−6^
Pyrene	4.93	4.88	0.22	0.14	1.23	1.25	8.19	8.80	5.434 × 10^4^	1.72 × 10^4^	1.19 × 10^−5^
Cyclopenta[c,d]pyrene	5.70	N/A	0.03	N/A	1.30	N/A	10.15	N/A	1.77 × 10^5^	8.84 × 10^4^	1.23 × 10^−7^
Benzo[a]anthracene	5.52	5.76	0.03	0.01	1.19	1.19	9.07	N/A	1.77 × 10^5^	9.97 × 10^4^	1.2 × 10^−5^
Chrysene	5.52	5.81	0.03	0.00	1.19	1.27	9.48	N/A	1.81 × 10^5^	1.10 × 10^5^	5.23 × 10^−6^
Benzo[b]fluoranthene	6.11	5.78	0.02	0.00	1.28	N/A	10.35	N/A	5.99 × 10^5^	1.04 × 10^5^	6.57 × 10^−7^
Benzo[k]fluoranthene	6.11	6.11	0.01	0.00	1.28	1.29	10.73	N/A	5.87 × 10^5^	2.01 × 10^5^	5.84 × 10^−7^
Benzo[j]fluoranthene	6.11	N/A	0.01	0.00	1.27	1.29	10.59	N/A	5.99 × 10^5^	2.01 × 10^5^	2.03 × 10^−7^ *
Benzo[a]pyrene	6.11	6.13	0.01	0.00	1.27	1.29	10.86	N/A	5.87 × 10^5^	2.088 × 10^5^	4.57 × 10^−7^
Benzo[e]pyrene	6.11	6.44	0.01	0.01	1.27	N/A	11.35	N/A	5.99 × 10^5^	3.88 × 10^5^	3 × 10^−7^
Perylene	6.11	6.25	0.01	0.00	1.28	1.29	10.07	N/A	5.99 × 10^5^	2.65 × 10^5^	3.65 × 10^−6^
Indeno[1,2,3-cd]pyrene	6.70	N/A	0.00	0.00	1.35	1.38	11.55	N/A	1.95 × 10^6^	6.52 × 10^5^	3.48 × 10^−7^
Dibenzo[a,h]anthracene	6.70	6.75	0.00	0.00	1.24	1.23	11.78	N/A	1.91 × 10^6^	4.74 × 10^5^	1.41 × 10^−7^
Benzo[b]chrysene	6.70	6.75	0.00	0.00	1.24	1.23	11.78	N/A	1.91 × 10^6^	4.74 × 10^5^	1.41 × 10^−7^
Benzo[g,h,i]perylene	6.70	6.63	0.00	0.00	1.35	N/A	11.50	N/A	1.95 × 10^6^	5.67 × 10^5^	3.31 × 10^−7^
Coronene	7.28	7.64	0.00	0.00	1.37	N/A	13.70	N/A	6.35 × 10^6^	4.27 × 10^6^	2.68 × 10^−9^ *
1-Indanone	2.11	N/A	1427.00	N/A	1.13	1.15	5.82	N/A	103.10	194.00	1.47 × 10^−6^ *
1-Acenaphthenone	2.79	N/A	20.03	N/A	1.21	N/A	7.63	N/A	611.40	461.20	3.51 × 10^−7^ *
1,2-Acenaphthylenedione	2.97	1.95	90.14	N/A	1.26	1.42	8.80	N/A	10.00	35.09	3.43 × 10^−9 +^
9-Fluorenone	3.55	3.58	3.74	N/A	1.19	1.24	8.14	N/A	1137.00	1262.00	6.77 × 10^−7 +^
2-Methyl-9-Fluorenone	4.10	N/A	1.15	N/A	1.17	N/A	8.62	N/A	1824.00	2446.00	7.47 × 10^−7 +^
1,8-Naphthalic anhydride	3.24	N/A	5.88	N/A	1.45	1.45	7.84	N/A	15.16	181.40	6.19 × 10^−7 +^
4-Hydroxy-9-Fluorenone	3.07	N/A	32.22	N/A	1.27	N/A	11.61	N/A	1490.00	967.50	7.05 × 10^−11 +^

**Table 3:** In silico prediction of the potential of toxicity of PAHs and related compounds using the TEST software.

PAHs	Fathead Minnow LC_50_ (96 h) (mg/L)	*D. magna* LC_50_ (48 h) (mg/L)	*T. pyriformis* IGC50 (48 h) (mg/L)	Oral Rat LC_50_ (mg/kg)	Bioconcentration Factor	Developmental Toxicity	Mutagenicity
Naphthalene	7.71	7.42	33.47	1411.67	244.67	0.32 (−)	N/A
Acenaphthylene	0.23	3.68	9.28	1177.63	379.92	0.71 (+)	N/A
Acenaphthene	1.71	2.35	18.62	1537.58	366.18	0.58 (+)	0.40 (−)
Fluorene	2.69	2.03	14.73	1754.56	614.66	0.63 (+)	0.37 (−)
Phenanthrene	0.89	0.82	9.61	1591.83	633.17	0.65 (+)	0.63 (+)
Anthracene	0.77	0.71	9.96	1549.94	614.34	0.64 (+)	0.60 (+)
Fluoranthene	0.24	0.71	4.66	993.93	481.32	0.74 (+)	0.84 (+)
Pyrene	0.22	0.51	3.86	1424.53	529.08	0.34 (−)	0.84 (+)
Cyclopenta[c,d]pyrene	2.14 × 10^−2^	0.30	1.25	N/A	685.78	0.66 (+)	1.00 (+)
Benzo[a]anthracene	0.12	0.27	2.28	2103.42	528.85	0.69 (+)	1.07 (+)
Chrysene	0.12	0.24	2.47	1996.76	556.81	0.63 (+)	1.04 (+)
Benzo[b]fluoranthene	4.86 × 10^−2^	0.15	0.87	N/A	605.33	0.50 (+)	1.04 (+)
Benzo[k]fluoranthene	5.51 × 10^−2^	0.15	0.87	N/A	483.79	0.50 (−)	1.03 (+)
Benzo[j]fluoranthene	4.89 × 10^−2^	0.16	0.87	N/A	550.26	0.49 (−)	0.95 (+)
Benzo[a]pyrene	3.18 × 10^−2^	0.20	0.87	N/A	569.11	0.37 (−)	1.02 (+)
Benzo[e]pyrene	5.35 × 10^−2^	0.19	0.87	N/A	654.33	0.36 (−)	0.98 (+)
Perylene	5.41 × 10^−2^	0.18	0.87	N/A	724.56	0.36 (−)	1.04 (+)
Indeno[1,2,3-cd] pyrene	N/A	7.58 × 10^−2^	0.53	N/A	665.05	N/A	1.04 (+)
Dibenzo[a,h]anthracene	4.27 × 10^−2^	0.11	0.54	434.97	587.54	0.37 (−)	0.94 (+)
Benzo[b]chrysene	4.28 × 10^−2^	0.15	0.54	474.17	592.05	0.37 (−)	0.84 (+)
Benzo[g,h,i]perylene	N/A	0.14	0.53	N/A	765.85	N/A	0.97 (+)
Coronene	N/A	7.53 × 10^−2^	0.36	N/A	910.01	N/A	1.09 (+)
1-Indanone	14.53	14.95	66.14	1243.69	8.70	0.46 (−)	0.40 (−)
1-Acenaphthenone	2.09	5.92	12.11	510.26	18.30	0.36 (−)	0.72 (+)
1,2-Acenaphthylenedione	1.30	13.02	5.59	262.55	8.36	0.30 (−)	0.85 (+)
9-Fluorenone	3.58	2.81	26.63	2192.00	75.84	0.64 (+)	0.50 (+)
2-Methyl-9-Fluorenone	2.21	1.80	17.94	1001.77	133.53	0.60 (+)	0.25 (−)
1,8-Naphthalic anhydride	N/A	N/A	N/A	2459.50	N/A	0.70 (+)	0.57 (+)
4-Hydroxy-9-Fluorenone	3.10	2.23	19.52	1068.02	63.01	0.82 (+)	0.50 (−)

**Table 4:** Molecular-docking results for the interaction between 4XNN and selected PAHs and related compounds.

Chemical Name	AC Score	SwissParam Score	Interaction	Residue
Phenalene	14.6297	−6.2712	Hydrophobic contacts	[THR]184, [MET]279, [VAL]265, [VAL]266, [TYR]182
Naphthalene	8.6925	−5.8241	Hydrophobic contacts	[THR]184, [MET]279, [VAL]265, [VAL]266, [TYR]182
Acenaphthylene	27.3166	−6.1687	Hydrophobic contacts	[THR]184, [MET]279, [VAL]265, [VAL]266, [TYR]182
Acenaphthene	18.6870	−6.1426	Hydrophobic contacts	[THR]184, [MET]279, [VAL]265, [VAL]266, [TYR]182
Fluorene	22.2790	−6.1441	Hydrophobic contacts	[THR]184, [VAL]265, [VAL]266
Phenanthrene	21.7864	−6.2722	Hydrophobic contacts	[MET]279, [VAL]265, [VAL]266, [TYR]182
Anthracene	23.5732	−6.3379	Hydrophobic contacts	[THR]255, [MET]279, [GLU]84
Fluoranthene	40.4039	−6.5404	Hydrophobic contacts	[THR]184, [MET]279, [VAL]265, [VAL]266, [TYR]182
Pyrene	26.6728	−6.447	Hydrophobic contacts	[THR]184, [MET]279, [VAL]265, [VAL]266, [TYR]182
Cyclopenta[c,d]pyrene	47.2204	−6.6178	Hydrophobic contacts	[THR]184, [VAL]265, [VAL]266, [TYR]182
Benzo[a]anthracene	47.1989	−6.6196	Hydrophobic contacts	[THR]184, [VAL]265, [VAL]266, [TYR]182
Chrysene	37.5780	−6.5771	Hydrophobic contacts	[THR]184, [MET]279, [VAL]265, [VAL]266, [TYR]182
Benzo[b]fluoranthene	52.2727	−6.7689	Hydrophobic contacts	[THR]255, [GLU]84, [ASN]379
Benzo[k]fluoranthene	49.0018	−6.7003	Hydrophobic contacts	[THR]255, [THR]184, [MET]279, [GLU]84, [LEU]210, [VAL]266
Benzo[j]fluoranthene	52.9186	−6.8241	Hydrophobic contacts	[THR]255, [THR]184, [VAL]266, [GLU]84, [LEU]210
Benzo[a]pyrene	39.4754	−6.8148	Hydrophobic contacts	[THR]184, [MET]279, [VAL]265, [VAL]266, [TYR]182
Benzo[e]pyrene	46.9974	−6.7042	Cation π interaction, Hydrophobic contacts	[GLU]84, [THR]184, [THR]255
Perylene	47.5805	−6.6342	Hydrophobic contacts	[GLU]84, [THR]184, [THR]255
Indeno[1,2,3-cd]pyrene	60.0970	−6.9315	Hydrophobic contacts	[THR]255, [THR]184, [GLU]84, [LEU]210, [VAL]266
Dibenzo[a,h]anthracene	42.3091	−6.9921	Hydrophobic contacts	[THR]255, [THR]184 [GLU]84, [LEU]210, [VAL]266
Benzo[b]chrysene	37.5780	−6.5771	Hydrophobic contacts	[THR]184, [MET]279, [VAL]265, [VAL]266, [TYR]182,
Benzo[g,h,i]perylene	51.2313	−6.8218	Hydrophobic contacts	[THR]255, [THR]184, [GLU]84
Coronene	56.5091	−6.8649	Hydrophobic contacts	[THR]87, [THR]184, [THR]255, [GLU]84
1-Indanone	4.5814	−5.8672	Hydrophobic contacts	[TYR]182, [VAL]266
1-Acenaphthenone	25.2732	−6.1708	Hydrogen bond, Hydrophobic contacts	[THR]184, [MET]279, [VAL]266, [TYR]182
1,2-Acenaphthylenedione	41.6846	−6.2234	Hydrogen Bond, Hydrophobic contacts	[THR]184, [MET]279, [TYR]182
9-Fluorenone	33.5335	−6.1484	Hydrogen bond, Hydrophobic contacts	[THR]184, [MET]279, [VAL]265 [VAL]266, [ASN]208
2-methyl-9H-fluoren-9-one	31.8207	−6.3944	Hydrogen bond, Hydrophobic contacts	[THR]184, [MET]279, [VAL]265, [VAL]266, [ASN]277
1,8-Naphthalic anhydride	17.6112	−6.4257	Hydrogen bonds, Hydrophobic contacts	[THR]184, [MET]279, [VAL]266
4-Hydroxy-9-Fluorenone	27.6711	−6.1791	Hydrogen bond, Hydrophobic contacts	[THR]184, [MET]279, [VAL]265, [VAL]266, [ASN]208

**Table 5:** The prediction of ADME properties.

PAHs	GI Absorption	BBB Permanent	P-gp Substatrate	CYP1A2 Inhibitor	CYP2C19 Inhibitor	CYP2C9 Inhibitor	CYP2D6 Inhibitor	CYP3A4 Inhibitor	Log Kp (Skin Permeation) (cm/s)	Lipinski	Bioavailability Score
Phenalene	Low	Yes	No	Yes	No	No	No	No	−4.30	Yes	0.55
Naphthalene	Low	Yes	No	Yes	No	No	No	No	−4.74	Yes	0.55
Acenaphthylene	Low	Yes	No	Yes	No	No	No	No	−4.43	Yes	0.55
Acenaphthene	Low	Yes	Yes	Yes	No	No	No	No	−4.46	Yes	0.55
Fluorene	Low	Yes	Yes	Yes	Yes	No	No	No	−4.35	Yes	0.55
Phenanthrene	Low	Yes	No	Yes	Yes	No	No	No	−4.22	Yes	0.55
Anthracene	High	Yes	No	Yes	Yes	No	No	No	−5.16	Yes	0.55
Fluoranthene	Low	No	No	Yes	No	No	No	No	−3.87	Yes	0.55
Pyrene	Low	No	No	Yes	No	No	No	No	−4.07	Yes	0.55
Cyclopenta[c,d]pyrene	Low	No	Yes	Yes	No	No	No	No	−3.76	Yes	0.55
Benzo[a]anthracene	Low	No	No	Yes	No	No	No	No	−3.60	Yes	0.55
Chrysene	Low	No	No	Yes	No	No	No	No	−3.57	Yes	0.55
Benzo[b]fluoranthene	Low	No	No	Yes	No	No	No	No	−3.74	Yes	0.55
Benzo[k]fluoranthene	Low	No	No	Yes	No	No	No	No	−3.50	Yes	0.55
Benzo[j]fluoranthene	Low	No	No	Yes	No	No	No	No	−3.50	Yes	0.55
Benzo[a]pyrene	Low	No	No	Yes	No	No	No	No	−3.50	Yes	0.55
Benzo[e]pyrene	Low	No	No	Yes	No	No	No	No	−3.27	Yes	0.55
Perylene	Low	No	No	Yes	No	No	No	No	−3.4	Yes	0.55
Indeno[1,2,3-cd]pyrene	Low	No	No	Yes	No	No	No	No	−3.45	Yes	0.55
Dibenzo[a,h]anthracene	Low	No	No	Yes	No	No	No	No	−3.21	Yes	0.55
Benzo[b]chrysene	Low	No	No	Yes	No	No	No	No	−2.95	Yes	0.55
Benzo[g,h,i]perylene	Low	No	No	Yes	No	No	No	No	−3.2	Yes	0.55
Coronene	Low	No	No	Yes	No	No	No	No	−2.71	Yes	0.55
1-Indanone	High	Yes	No	No	No	No	No	No	−5.92	Yes	0.55
1-Acenaphthenone	High	Yes	No	Yes	No	No	No	No	−5.47	Yes	0.55
1,2-Acenaphthylenedione	High	Yes	No	Yes	Yes	No	No	No	−6.03	Yes	0.55
9-Fluorenone	High	Yes	No	Yes	Yes	No	No	No	−4.86	Yes	0.55
2-methyl-9H-fluoren-9-one	High	Yes	No	Yes	Yes	No	No	No	−4.69	Yes	0.55
1,8-Naphthalic anhydride	High	Yes	No	Yes	No	No	No	No	−5.91	Yes	0.55
4-Hydroxy-9-Fluorenone	High	Yes	No	Yes	No	No	No	No	−5.58	Yes	0.55

## Data Availability

Data will be made available upon request.
